# 

**Published:** 1886-01

**Authors:** 


Whole Number, 313. JANUARY, 1886. Vol. LII., Number 1.

### THE CHICAGO MEDICAL JOURNAL AND EXAMINER

EDITORS:

S. J. JONES, M.D., LL.D.

W. W. JAGGARD, A.M., M.D. HAROLD N. MOYER, M.D.

COLLABORATORS.

CHICAGO:

CHICAGO:

PUBLISHED by CLARK & LONGLEY, under direction of THE CHICAGO MEDICAL PRESS ASSOCIATION,

240 WABASH AVENUE.

